# Mechanism of Wuzhuyu decoction on alcohol-induced gastric ulcers using integrated network analysis and metabolomics

**DOI:** 10.3389/fphar.2023.1308995

**Published:** 2024-01-08

**Authors:** Xin Wang, Lisheng Chen, Lei Chang, Yong He, Tingting He, Ruilin Wang, Shizhang Wei, Manyi Jing, Xuelin Zhou, Haotian Li, Yanling Zhao

**Affiliations:** ^1^ College of Pharmacy, Chengdu University of Traditional Chinese Medicine, Chengdu, China; ^2^ Department of Pharmacy Department, Fifth Medical Center of Chinese PLA General Hospital, Beijing, China; ^3^ College of Pharmacy, Southern Medical University, Guangzhou, China; ^4^ Integrative Medical Center, The Fifth Medical Center of Chinese PLA General Hospital, Beijing, China; ^5^ Department of Pharmacology, School of Basic Medical Sciences, Capital Medical University, Beijing, China

**Keywords:** Wuzhuyu decoction, gastric ulcer, network analysis, metabolomics, alcohol

## Abstract

**Background:** Gastric ulcers (GUs) are prevalent digestive disorders worldwide. Wuzhuyu Decoction (WZYT) is a traditional Chinese medicine that has been employed for centuries to alleviate digestive ailments like indigestion and vomiting. This study aims to explore the potential effects and underlying mechanisms of WZYT on alcohol induced gastric ulcer treatment.

**Methods:** We employed macroscopic assessment to evaluate the gastric ulcer index (UI), while the enzyme-linked immunosorbent assay (ELISA) was utilized for detecting biochemical indicators. Pathological tissue analysis involved hematoxylin-eosin (H&E) staining and Periodic Acid-Schiff (PAS) staining to assess gastric tissue damage. Additionally, the integration of network analysis and metabolomics facilitated the prediction of potential targets. Validation was conducted using Western blotting.

**Results:** The research revealed that WZYT treatment significantly reduced the gastric ulcer index (UI) and regulation of alcohol-induced biochemical indicators levels. Additionally, improvements were observed in pathological tissue. Network analysis results indicated that 62 compounds contained in WZYT modulate alcohol-induced gastric ulcers by regulating 183 genes. The serum metabolomics indicated significant changes in the content of 19 metabolites after WZYT treatment. Two pivotal targets, heme oxygenase 1 (HMOX1) and albumin (ALB), are believed to assume a significant role in the treatment of gastric ulcers by the construction of “compounds-target-metabolite” networks. Western blot analysis confirmed that WZYT has the capacity to elevate the expression of HMOX1 and ALB targets.

**Conclusion:** The integration of network analysis and metabolomics provides a scientific basis to propel the clinical use of WZYT for GUs. Our study provides a theoretical basis for the use of Wuzhuyu decoction in the treatment of gastric ulcers.

## Graphical Abstract

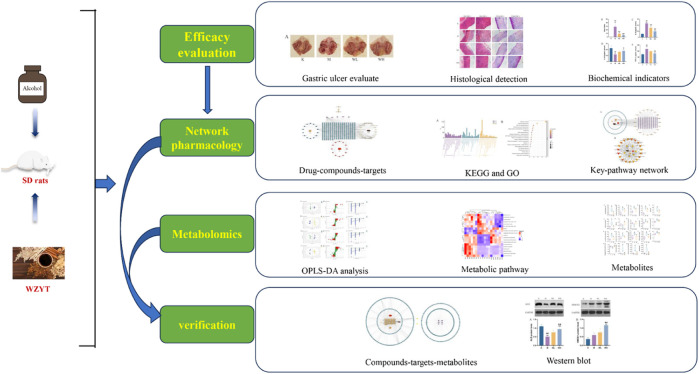

## 1 Introduction

Gastric ulcer is a common digestive disorder, affecting 5%–10% of the world’s population (Muazzam et al., 2021). The occurrence of this disease is influenced by an imbalance of aggressive factors, including poor diet, physical stress, tobacco abuse, prolonged administration of aspirin or other non-steroidal anti-inflammatory drugs (NSAIDs), excessive caffeine intake, and *Helicobacter pylori* infection (Zhang et al., 2022a). Alcohol consumption is a significant contributor to the development of gastric ulcers. Research has revealed that alcohol directly affects the gastric mucosa, increases permeability, triggers necrosis, and exacerbates inflammation by intensifying oxidative stress reactions (Wang et al., 2020). Patients often face delayed treatment due to rapid disease progression, leading to gastric hemorrhage (Rahman et al., 2020; Hu et al., 2021). The incidence of gastric ulcers significantly impacts the quality of life, therefore, effective treatment is crucial.

The current mainstream drug on the market for the treatment of gastric ulcers is a proton-pump inhibitor, and research revealed the mechanism of this medicine action primarily from suppressed gastric acid secretion (Ward and Kearns, 2013; Wedemeyer and Blume, 2014). Proton-pump inhibitor drugs remarkably alleviate the pain of alcohol-induced gastric ulcers, but long-term use could lead to severe adverse reactions such as headache, diarrhea, and risk of intestinal flora infection (Thomson et al., 2010; Yu et al., 2017). Considering the aforementioned reasons, the pursuit of a novel drug with minimal side effects is increasingly crucial.

Traditional Chinese medicine is a great treasure of the Chinese people, and recently more research revealed the curative effect on various causes of gastric ulcers (Song et al., 2020; Li et al., 2021a; Lu et al., 2021). From the perspective of traditional Chinese medicine to treatment of diseases in the digestive system is becoming a novelty aspect. The main components of Wu-Zhu-Yu decoction (WZYT) are four herbs: *Tetradium ruticarpum* (A.Juss.) T.G.Hartley (wzy), *Zingiber officinale* Roscoe (sj), *Panax ginseng* C.A.Mey (rs), and *Ziziphus jujuba* Mill (dz). The earliest record of this medicine can be traced back to Shang-Han-Lun, where it was utilized to address headaches and gastrointestinal disorders thousands of years ago (Hu et al., 2012; Liu et al., 2017). Historical experience has shown that the combination of the four herbs greatly exploits the advantages of Chinese herbal medicine (Nan et al., 2022). Prior research has validated the distinctive efficacy of WZYT in the management of chronic migraine, non-alcoholic fatty liver disease, and liver cancer (Zhang et al., 2022b; Nan et al., 2022; Ouyang et al., 2023). It has also been shown anti-inflammatory effects and to be effective in the treatment of gastric ulcers (Odaguchi et al., 2006; Cai et al., 2023). Modern pharmacological studies also found that many of the compounds in WZYT are useful in digestive disease such as berberine, dehydroevodiamine, rutaecarpine, and so on (Al-Qarawi et al., 2005; Shin et al., 2020; Yang et al., 2020; Wen et al., 2021). However, whether WZYT has an effect on alcohol-induced gastric ulcers is unclear.

Network analysis provides strategies for the study of multiple-component TCM systems. Growing evidence that network analysis can make predictions for herbal component-specific disease interactions (Li et al., 2022a). Metabolomics is a technique employed for identifying global changes in the metabolic profiles of organisms under both normal physiological and pathological conditions (Pan et al., 2020). The integration of network analysis and metabolomics offers robust evidence for elucidating the mechanism of specific diseases (Zhang et al., 2020). Here, we hypothesize that WZYT is effective in the prevention of gastric ulcers, and further predict the possible mechanisms by integrating network analysis and metabolomics.

## 2 Materials and methods

### 2.1 Regents

The *T. ruticarpum* (A.Juss.) T.G.Hartley, *Z. officinale* Roscoe, *P. ginseng* C.A.Mey, and *Z. jujuba* Mill were purchased from Lvye Pharmaceutical Co., Ltd. (Beijing, China) (Cat: 21030801, 22041904, 21112603, 21081801) and the plants were identified and authenticated through microscopic identification and thin layer chromatography (TLC) by company according to the Chinese Pharmacopoeia (2020). The enzyme-linked immunosorbent assay kits of IL-6, IL-10, and TNF-α were purchased from Shanghai Enzyme-linked Biotechnology Co., Ltd. (Cat: ml064292; ml002813; ml002859). The primary antibodies of albumin (ALB) (1: 1,000), and Heme Oxygenase 1 (HMOX1) (1: 1,000) were obtained from Wuhan Sanying Biotechnology Co., Ltd. (Cat: 16475-1-AP; 10701-1-AP).

### 2.2 Preparation of WZYT

The preparation of WZYT followed methodologies outlined in prior literature with minor modifications (Nan et al., 2022). The four medicines, *T. ruticarpum* (A.Juss.) T. G. Hartley (wzy), *Z. officinale* Roscoe (sj), *P. ginseng* C. A. Mey. (rs), and *Z. jujuba* Mill. (dz), were soaked in water for 30 min, after which eight times the amount of water was added, and the mixture was brought to a boil. Subsequently, the heating was continued for an additional 30 min. Pour out the filtrate, and add six times the volume of water to repeat the aforementioned process. Afterward, collect all the filtrate, and proceed with rotary evaporation and freeze-drying to obtain a final medicine. Each 1 g of powder contains 2.75 g of the crude drug, converted to the amount of drug given by the original herb. Analysis of the quality characteristics of the WZYD was consistent with previous literature (Hu et al., 2023). Store the powder at 4°C. Before administration, ensure the medicine is brought to room temperature and dissolved in distilled water to achieve the required dose.

### 2.3 Animal groups and experimental design

**FIGURE 1:**
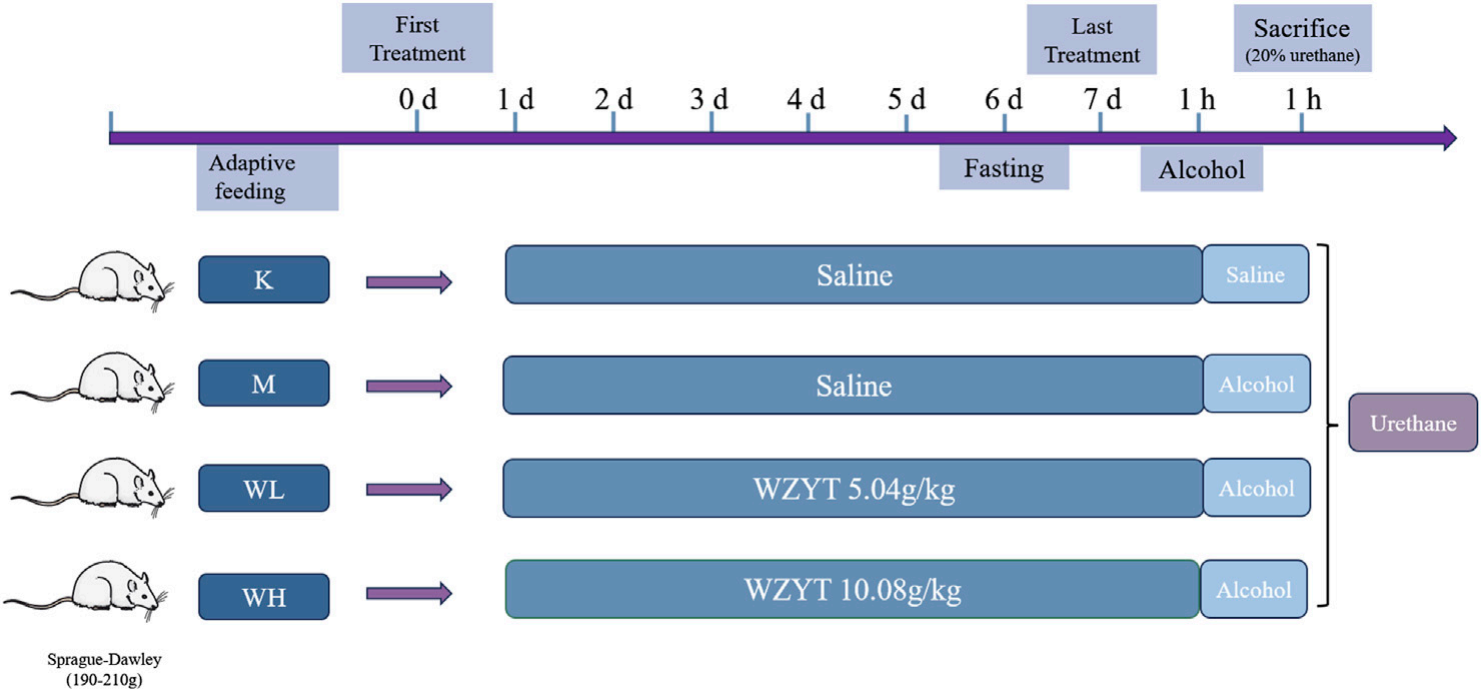
Flow chart of drug administration in animal experiments.

Twenty-four male Sprague-Dawley rats weighing 190 g–210 g were procured from the Beijing Sibeifu Animal Breeding Centre. The animals were acclimated for 5 days under controlled conditions: temperature of 25°C ± 0.5°C, relative humidity of 55% ± 5%, and a 12-h light-dark cycle. During the acclimation period, food and water were provided *ad libitum*. After acclimation, all the rats were randomly assigned to four groups, each containing six rats. The low-dose (WL) group received 5.04 g/kg of WZYT, while the high-dose (WH) group received 10.08 g/kg of WZYT (crude drug/body weight) via gavage for 7 days. The dosage selection was based on previous references and slightly adjusted in actual experiments (Xu et al., 2016; Liu et al., 2017; Nan et al., 2022). The control (K) and model (M) groups were administered equal volumes of distilled water by gavage. One hour after the final gavage, all rats, except those in the control group, were gavaged with absolute alcohol at 5 mL/kg to induce gastric ulcers (Balan et al., 2015). After 60 min, all animals were anesthetized by intraperitoneal injection of 20% urethane (injection dose of 5 mL/kg) (Figure 1). Blood was collected from the rat’s abdominal aorta and then centrifuged at 3,000 rpm for 10 minutes. The supernatant was collected and stored at −80°C for subsequent analysis.

### 2.4 Macroscopic assessment of gastric ulcer

The stomachs were excised and incised along the greater curvature. The gastric surface was then cleaned of debris using saline and gently dried with gauze. Subsequently, the stomachs were placed on coordinate paper and photographed. The ulcer area was calculated by ImageJ software, and the assessment of the gastric ulcer index (UI) was conducted in accordance with prior literature (Guzmán-Gómez et al., 2018).

$$UI=\frac{Ulcer\;mucosal\;area\left({mm}^{2}\right)}{Total\;mucosal\;area\left({mm}^{2}\right)}\times 100$$

### 2.5 Measurement of critical serum indicators

In order to detect the effect of WZYT on alcohol-induced gastric ulcers, the critical indicators interleukin-6 (IL-6), interleukin-10 (IL-10), and tumor necrosis factor-α (TNF-α) were measured according to the manufacturer’s instructions.

### 2.6 Pathological histological analysis

After the rats were anesthetized, a part of the gastric tissue was cut off and immersed in 4% paraformaldehyde. After 24 h of infiltration, the tissue was dehydrated using graded concentrations of alcohol and then submerged in paraffin wax. The embedded sections were cut to 4–5 μm and stained with hematoxylin and eosin (H&E) as well as periodic acid–schiff (PAS). Eventually, the sections were placed under a microscope for observation. The pathological injury index in each sample was graded from 0 to 4 (0, no pathological damage; 1, capillary hyperemia of lamina propria; 2, necrotic of mucosal epithelial cells, inflammatory cell infiltration; 3, formation of superficial erosion; 4, exacerbation of inflammation and ulcer formation).

### 2.7 Network analysis

#### 2.7.1 Collection of possible compounds and related targets

The main components and targets of WZYT were obtained by searching the Traditional Chinese Medicine Systems Pharmacology database (TCMSP) (https://old.tcmsp-e.com/tcmsp.php) and Swiss Target Prediction (http://www.swisstargetprediction.ch/) database. The screening criteria were set as oral bioavailability (OB) ≥ 30, and drug similarity (DL) ≥ 0.18. All targets were translated into the criteria gene names using the Uniport database (https://www.uniprot.org/). For gastric ulcers, we conducted a search in the Genecards database (https://www.genecards.org/) and OMIM database (https://omim.org/) to collect all the relevant targets.

#### 2.7.2 Protein-protein interaction (PPI) network construction

The Venn diagram (https://bioinfogp.cnb.csic.es/tools/venny/index.html) was employed to identify the common targets present at the intersection of components and disease. Then the common targets were input into the String database (https://www.string-db.org/) to get the protein-protein interaction (PPI) network. For the parameterization of the organism, we chose ‘*Homo sapiens*’ and the minimum required interactive score is set to 0.9 as in previous studies (He et al., 2022). All of the data was visualized using Cytoscape 3.9.1 software.

#### 2.7.3 GO and KEGG analysis

To further analyze the biological process of WZYT, we chose the DAVID database (https://david.ncifcrf.gov/) for Gene ontology (GO) and Kyoto Encyclopedia of Genes and Genomes (KEGG) analysis. GO is composed of three parts: biological process (BP), cellular components (CC), and molecular functions (MF). BP represents a specific overall biological process, CC indicates its primary location, and MF is the function at the molecular level. KEGG analysis displays associated pathways and mechanisms. All results were uploaded to the Bioinformatics platform (http://www.bioinformatics.com.cn/) for visual analysis.

### 2.8 Serum metabolomics analysis

#### 2.8.1 Preparation of serum samples

Frozen serum was removed from −80°C and thawed at room temperature. One hundred microliters of serum were added to three times the volume of pre-cooled methanol, vortexed for 1 min, and used for the assay.

#### 2.8.2 Chromatographic and mass spectrometric conditions

The ACQUITY UPLC^®^ HSS T3 C18 chromatography column (2.1 mm × 100 mm, 1.8 μm) was used. The mobile phase consisted of 0.1% formic acid in water (A) and 0.1% formic acid solution in acetonitrile (B). Gradient elution will be employed in this experiment. The elution gradient will run over a period of 16 min with the following proportions of mobile phase B: 5% for 0–6.0 min, 45% for 6.0–8.0 min, 75% for 8.0–12.0 min, 85% for 12.0–12.5 min, 100% for 12.5–14.0 min, 100%–5% for 14.0–14.5 min, and finally 5% from 14.5 to 16.0 min. The column temperature will be maintained at 40°C. The flow rate was 0.3 mL/min and the injection volume was 3 µL.

ESI source, data acquisition in MSE Continuum mode. The locking mass of the Leucine Enkephalin (ESI- *m/z* at 554.2615, ESI + *m/z* at 556.2771) solution was used for accurate mass determination. Capillary voltage: ESI- 2.5 kV, ESI+ 3.0 kV, ion source temperature: 140°C, desolvation temperature: 450°C, taper hole voltage: 40 V, taper hole gas flow rate: 50 L · h^−1^, desolvation flow rate: 800 L · h^−1^, collision energy: 10–45 V, scanning interval time 0.2 s. Mass scanning range: 50–1,200 m/z.

#### 2.8.3 Data processing and analysis

The raw data was extracted by the software Progenesis QI 2.4 and all data was saved in CSV format for further analysis. The data underwent normalization using MetaboAnalyst 5.0 (https://www.metaboanalyst.ca/). Subsequently, data were imported into SIMCA 14.1 for both principal component analysis (PCA) and orthogonal-partial least squares discriminant analysis (OPLS-DA). The metabolites that met VIP>1, p|(corr)| ≥ 0.58, *t*-test *p* < 0.5 were considered as potential biomarkers. The parameters are established based on prior references (Tong et al., 2021). All of the biomarkers were identified by the Human Metabolome Database (HMDB) (http://www.hmdb.ca/). Subsequently, the biomarkers’ KEGG IDs were entered into the MetaboAnalyst 5.0 for metabolic pathway analysis.

### 2.9 Integrated analysis of metabolomics and network analysis

The targets of biomarkers were obtained from Mbrole 2.0 (http://csbg.cnb.csic.es/mbrole2/). Subsequently, all target IDs were converted to standardized gene names using the Uniprot (https://www.uniprot.org/) database. Finally, the “Compounds-targets-metabolic” network was constructed by Cytoscape 3.9.1.

### 2.10 Mechanism analysis by western blot

A portion of stomach tissue was excised and nine times the volume of RIPA lysate mixed with PMSF was weighed and homogenized. The supernatant was quantified by BCA and added to the appropriate loading buffer. After electrophoresis, the samples were transferred to polyvinylidene difluoride (PVDF) membranes, then closed with 5% skimmed milk powder for 2 h. After TBST washing, the membranes were incubated with primary antibody overnight at 4°C, repeated washing with TBST, and incubated with secondary antibodies for 1 h at room temperature. Finally, ECL was added for development and the optical density was analyzed with ImageJ software.

### 2.11 Statistical analysis

The experimental data were analyzed by SPSS and visualized with GraphPad Prism software. One-way ANOVA was used as the analysis method, and all data was represented by mean ± standard deviation. *p*-value < 0.05 was considered statistically significant, *p*-value < 0.01 was considered highly statistically significant.

## 3 Results

### 3.1 Macroscopic assessment of gastric mucosal

**FIGURE 2:**
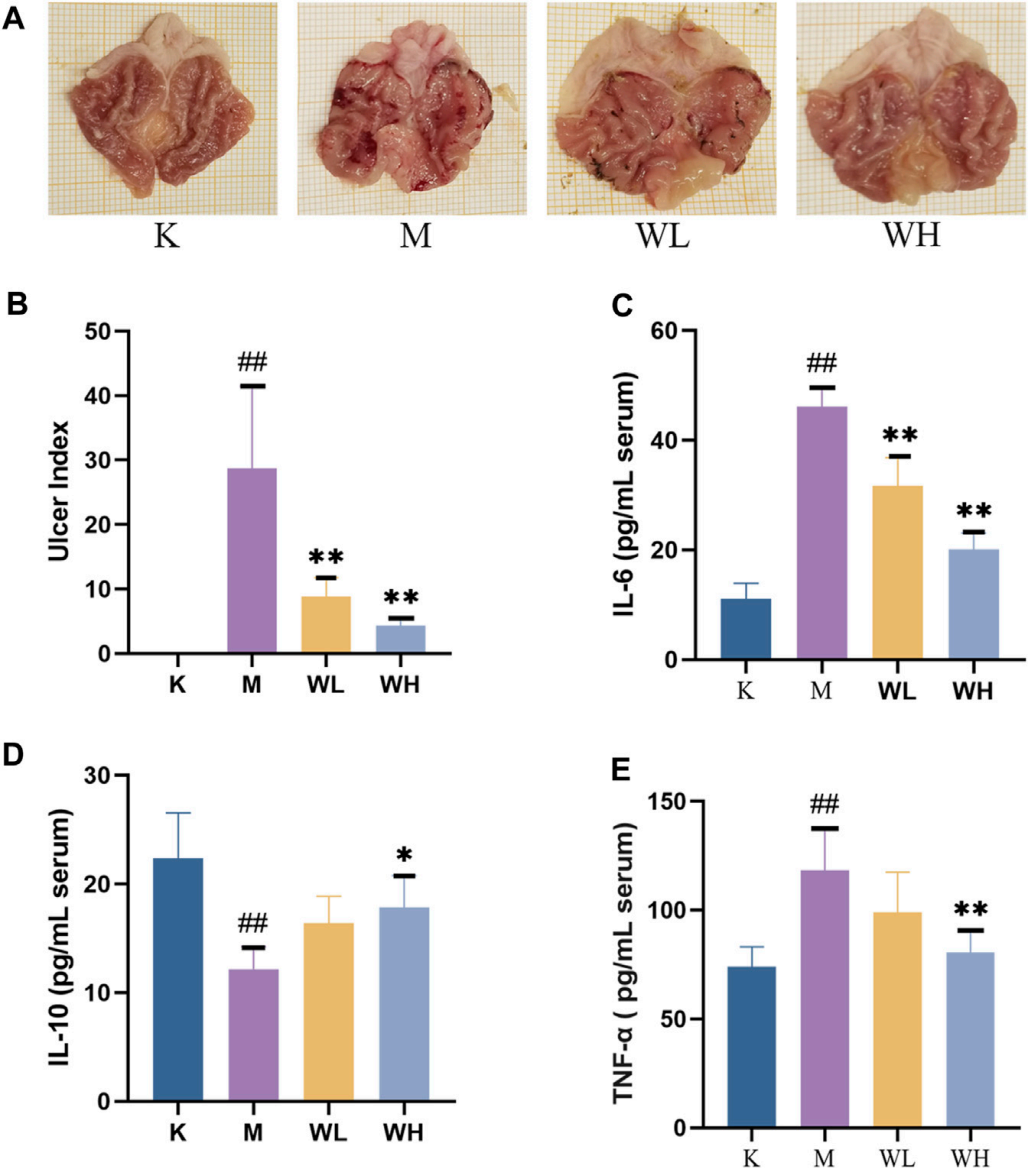
The effect of WZYT on alcohol-induced gastric ulcer. **(A)** Macroscopic assessment of gastric ulcer. **(B)** The effect of WZYT on UI of different groups. **(C–E)** Expression of IL-6, IL-10, and TNF-α in serum respectively. Data are presented as the mean ± SD (*n* = 6). ^#^ *p* < 0.05, ^##^ *p* < 0.01 compared with the K group; *p < 0.05, **p < 0.01 compared with the M group.

Compared to the M group, the K group gastric mucosa showed integrity and smooth surface with no hemorrhages. Alcohol administration leads to severe gastric lesions with hemorrhagic erosion, gastric mucosal integrity disruption, mucosal congestion, and swelling. The WL group gastric mucosal damage was significantly reduced, and bleeding in strips, and mucosal swelling had a significant improvement. The WH group gastric mucosal improvement was more pronounced (Figure 2A). In addition, we measured the gastric ulcer index among different groups, the results indicated that WZYT was able to dramatically reduce UI levels (Figure 2B).

### 3.2 Effect of WZYT on IL-6, IL-10, and TNF-α

Enzyme-linked immunosorbent assay (ELISA) was employed to quantify IL-6, IL-10, and TNF-α levels in the serum. The results revealed elevated levels of IL-6, TNF-α, and decreased levels of IL-10 after alcohol administration. However, WZYT exhibited the capacity to reverse these indicators (Figures 2C–E).

### 3.3 Effect of WZYT on histological

**FIGURE 3:**
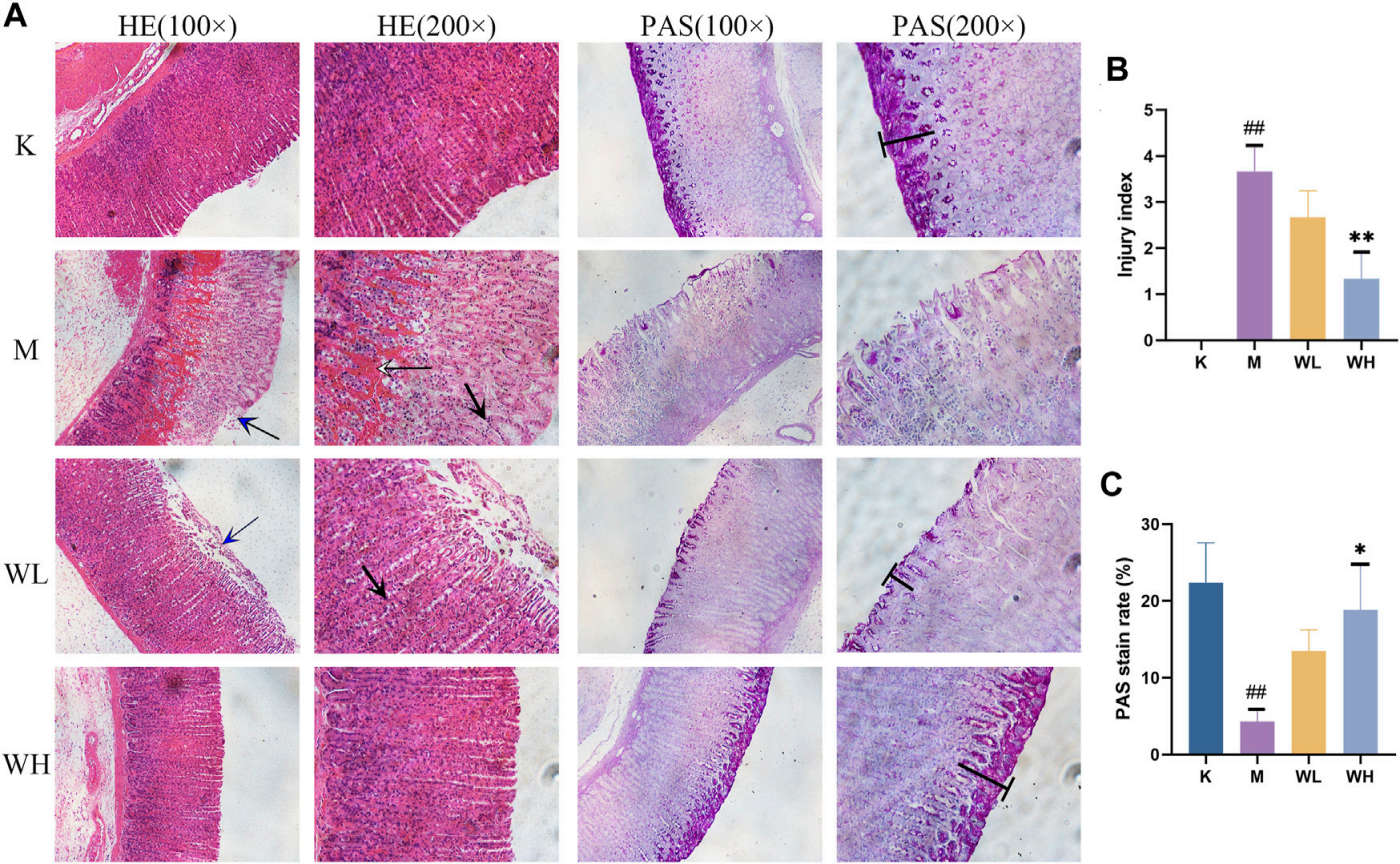
**(A)** The effects of WZYT on pathological changes in different groups. HE: stained with hematoxylin and eosin. PAS: stained with periodic acid–Schiff (×100 and ×200 magnification). **(B)** HE score of gastric injury index. **(C)** PAS positive rate. Data are presented as the mean ± SD (n = 3). ^#^ *p* < 0.05, ^##^ *p* < 0.01 compared with the K group; *p < 0.05, **p < 0.01 compared with the M group. Blue arrow: Epithelial cell shedding; Black arrow: infiltration of inflammatory cells; White arrow: hemorrhage of gastric tissue. Т: apical staining.

As shown in Figure 3A, the gastric mucosal glands of the K group displayed an intact and closely packed structure. Alcohol administration resulted in epithelial cell shedding, significant infiltration of inflammatory cells, and hemorrhage, the injury index has a significant increase (Figure 3B); Both the WL and WH groups exhibited pathological improvement. The WL group did not show significant hemorrhage compared to the M group. Additionally, PAS staining indicated a considerable decrease in apical staining due to alcohol, the staining rate had also had a significant reduction. Notably, WZYT enhanced apical staining intensity, with the WH group showing a more pronounced positive rate than the WL group (Figure 3C). The above results indicate that WZYT effectively improves gastric histopathology.

### 3.4 Network analysis results

#### 3.4.1 Compounds and target prediction

**FIGURE 4:**
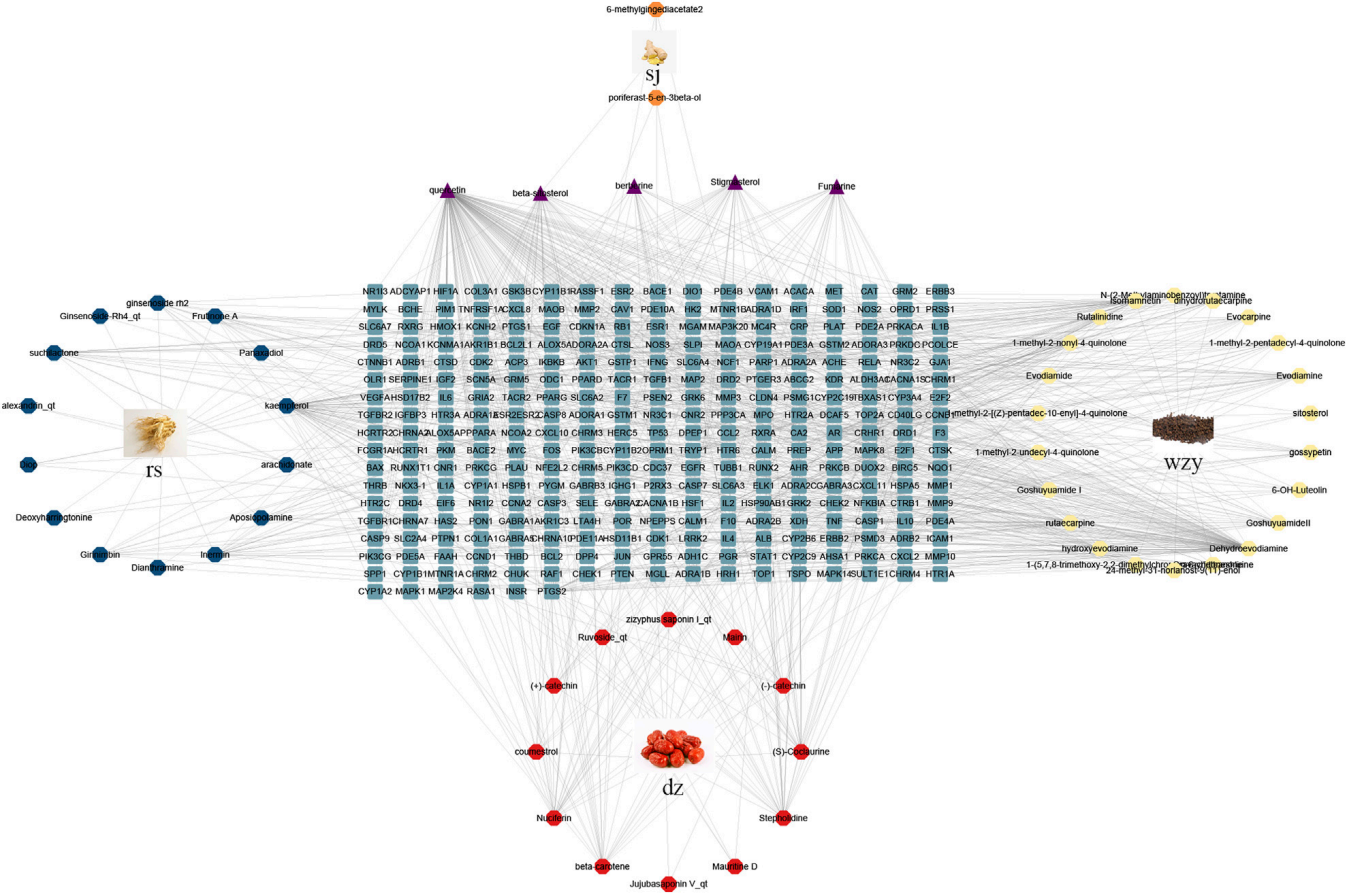
The “drug-compounds-targets” network. Yellow circle represents compounds of wzy; Red circle represents compounds of dz; Blue circle represents compounds of rs; Orange circle represents compounds of sj; Purple represent the common compounds; Blue squares represent different genes.

Employing information from both the TCMSP database and the Swiss Target Prediction database, we compiled a total of 62 compounds. Specifically, wzy contributes to 25 components, rs to 17 components, sj to 3 components, and dz to 17 components. After removing duplicates and translating to crucial gene names using the UniProt database, we acquired a total of 295 gene targets. The “drug-compounds-targets” network is depicted in Figure 4.

#### 3.4.2 Disease target collection and prediction

**FIGURE 5:**
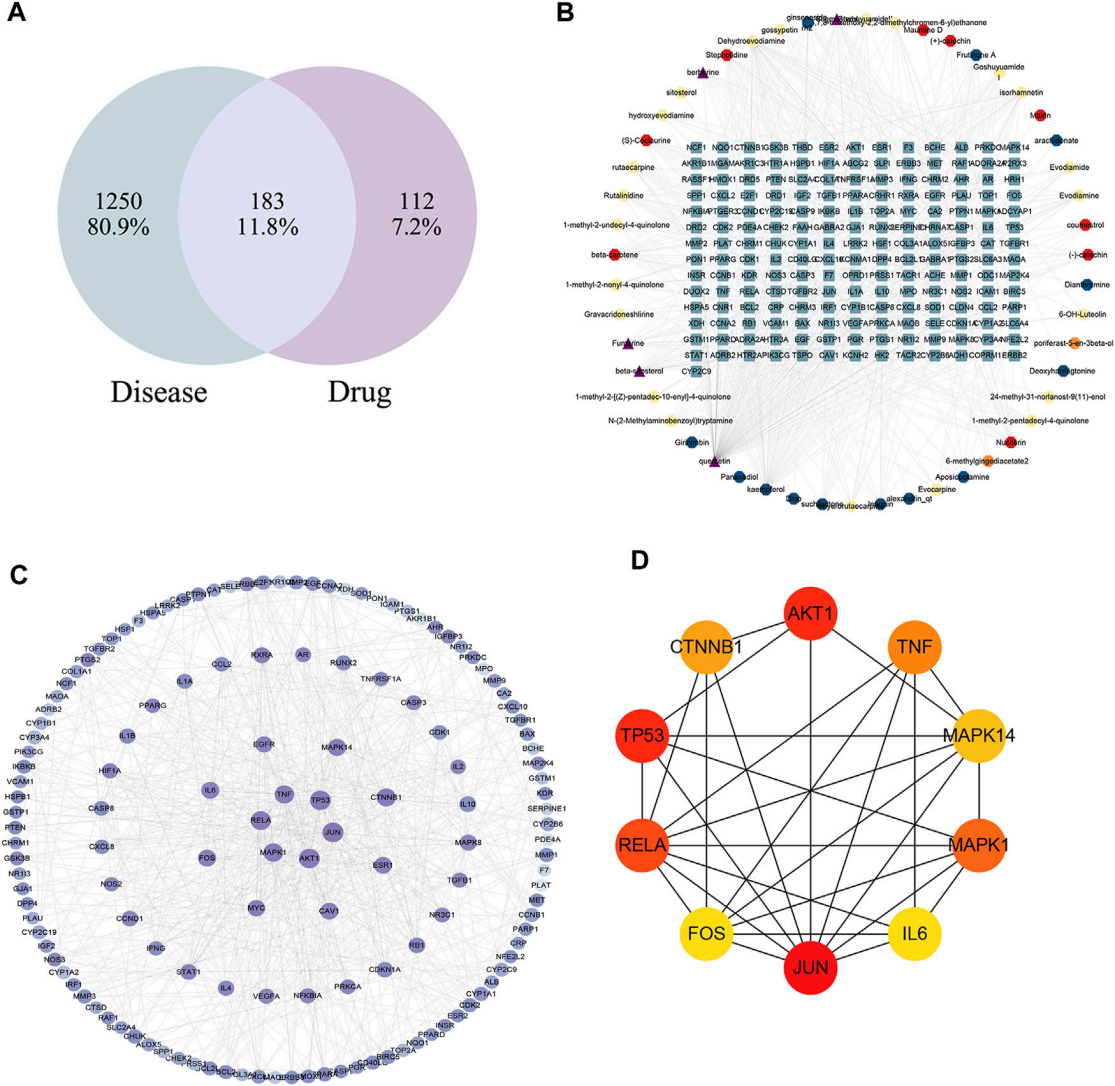
**(A)** Venn diagram of disease and WZYT. **(B)** “Compounds-target-disease” network. Different colors come from different drugs. **(C)** protein-protein interaction(PPI) network. **(D)** The top 10 targets of the PPI network.

The disease targets of alcohol-induced gastric ulcers were screened from Genecard and OMIM databases. In the Genecard database, we got 1351 gene targets; In the OMIM database, we got 114 gene targets. A total of 1433 gene targets were obtained after de-duplication. Then after the Venn database screen, 183 common targets were obtained (Figure 5A). To better visualize the relationship between the active compounds and the disease target, we established the “compounds-target-disease” network as shown in Figure 5B. The top 3 compounds ranked by degree are displayed in Table 1.

**TABLE 1:** Information of the top 3 compounds ranked by degree.

Molecular structure	Compound	Degree	Average Shortest Path Length	Betweenness Centrality	ClosenessCentrality	Herbs	CAS
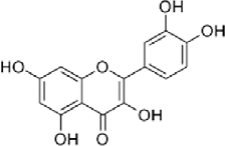	Quercetin	167	1.91	0.53	0.52	*wzy*	117-39-5
						*dz*	
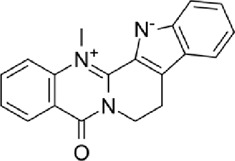	Dehydroevodiamine	97	2.30	0.36	0.43	*wzy*	67909-49-3
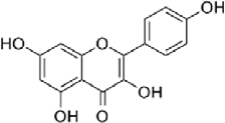	Kaempferol	61	2.52	0.08	0.40	*rs*	520-18-3

#### 3.4.3 PPI network instruction

The String database was used to investigate the protein-protein interaction (PPI) network. The common 183 targets were entered into the String database, organisms were selected as “Homo sapiens,” and the minimum required interaction score was set as the highest confidence 0.9. The TSV file was downloaded and further visualized using Cytoscape 3.9.1. The color and size of the nodes were sorted by degree. As shown in Figure 5C, the darker the color, the higher the degree. The top 10 targets of JUN, AKT1, TP53, RELA, MAPK1, TNF, CTNNB1, MAPK14, FOS, IL6 were identified as core targets (Figure 5D).

#### 3.4.4 Gene ontology (GO) and kyoto encyclopedia of genes and genomes (KEGG) enrichment analysis

**FIGURE 6:**
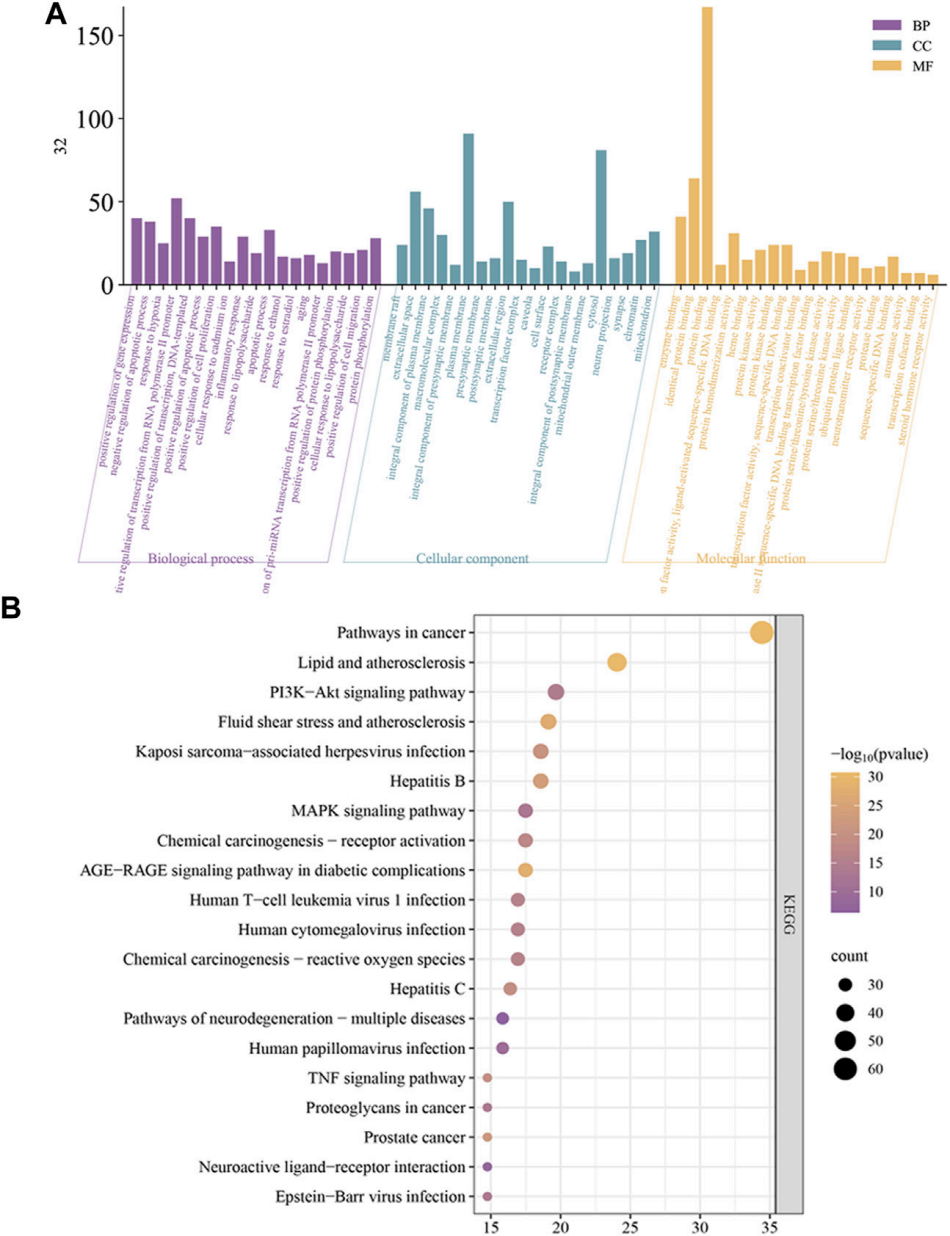
**(A)** GO enrichment analysis. **(B)** KEGG enrichment analysis.

The results of the GO analysis are displayed in Figure 6A. GO analysis showed the relevant biological processes including positive regulation of gene expression, response to hypoxia, and inflammatory response. Cellular components include extracellular space, plasma membrane, and cell surface. Molecular functions include enzyme binding, transcription factor binding, and nitric oxide synthase regulator activity. For pathways analysis, a total of 173 pathways were obtained, and the top 20 KEGG analysis enrichment results show related mechanisms involving Pathways in cancer, Lipid and atherosclerosis, and PI3K-Akt signaling pathway (Figure 6B).

#### 3.4.5 Pathway-targets network construction

**FIGURE 7:**
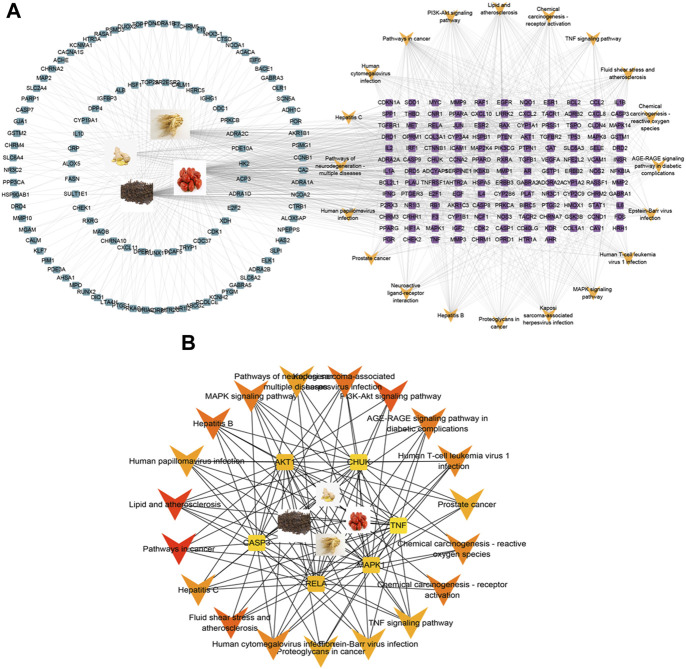
**(A)** “pathway-targets” network of the top 20 pathways. Blue squares represent targets of WZYT, Purple targets represent common targets of WZYT and pathway. Yellow “V” represent the top 20 pathways. **(B)** “key-targets” network analyzed by cytoHUBBa.

To gain a more profound understanding of the potential mechanism through which WZYT influences alcohol-induced gastric ulcers, the top 20 pathways and their enriched targets were constructed into a “pathway-targets” network (Figure 7A). Subsequently, the “key-targets” network was subjected to further analysis using cytoHUBBa. The MCC analysis findings highlighted the pivotal roles of the PI3K-AKT pathway in the therapeutic approach for alcohol-induced gastric ulcers (Figure 7B).

### 3.5 Serum metabolic analysis

#### 3.5.1 Multivariate statistical analysis

**FIGURE 8:**
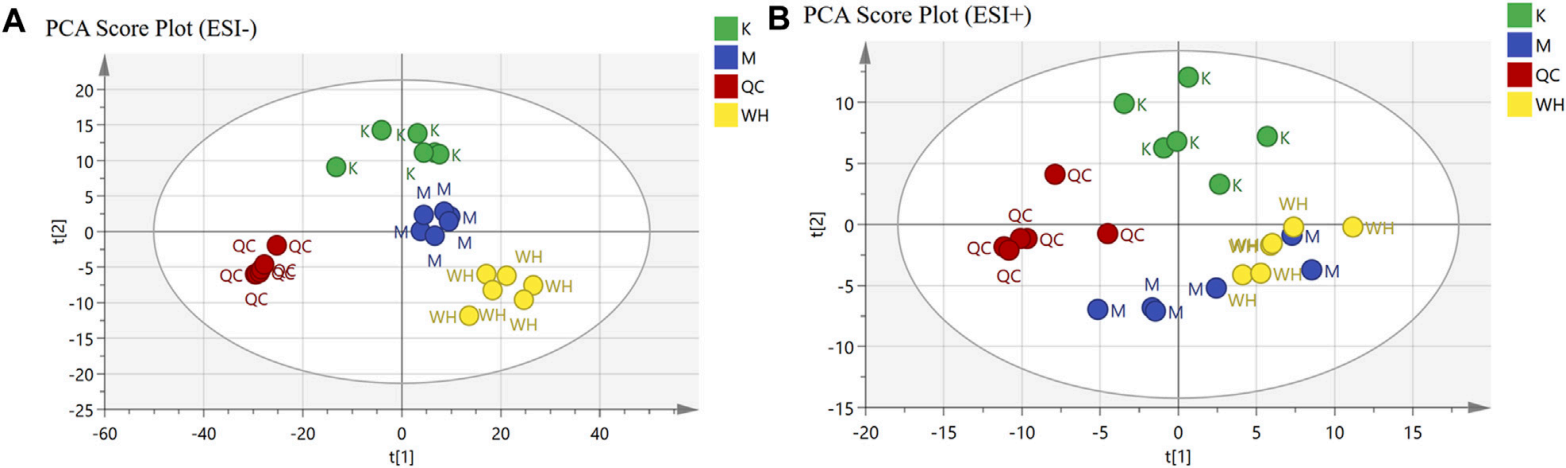
PCA analysis of different groups both in ESI- and ESI+ mode. **(A)**: ESI- mode. **(B)**: ESI+ mode.

Non-targeted serum metabolomics analysis was conducted on rats from various groups. Principal Component Analysis (PCA) analysis was employed to discern clustering trends among the K, M, and WH groups. Quality control (QC) was applied to evaluate sample stability. The PCA results revealed that there was significant clustering in the negative ion mode; however, the clustering trend in the positive ion mode was not significant in the M and WH groups (Figure 8).

**FIGURE 9:**
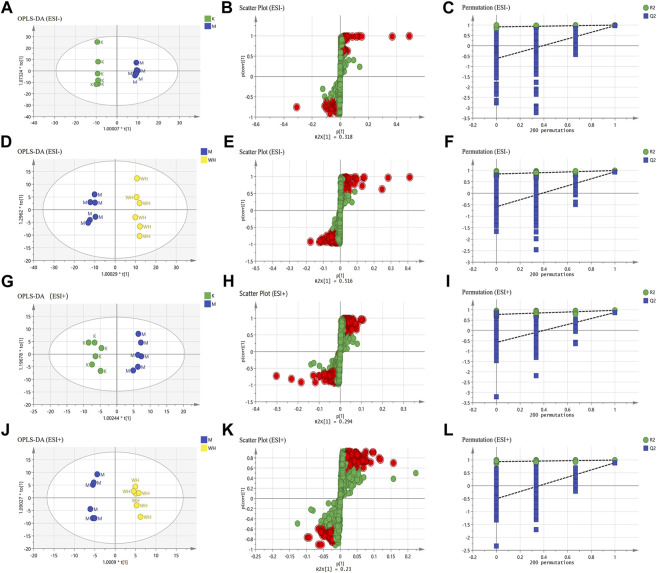
Results of serum metabolomics analysis. **(A,G)** The OPLS-DA analysis of the K group and the M group in ESI- and ESI+ mode. **(D,J)** The OPLS-DA analysis of the M group and the WH group in ESI- and ESI+ mode. **(B,H)** S-plot analysis of the K group and the M group in ESI- and ESI+ mode. **(E,K)** S-plot analysis of the M group and the WH group in ESI- and ESI+ mode. **(C,I)** 200 permutation tests of the K group and the M group in ESI- and ESI+ mode. **(F,L)** 200 permutation tests of the M group and the WH group in ESI- and ESI+ mode.

Orthogonal Projections to Latent Structures Discriminant Analysis (OPLS-DA) analysis was performed to identify differential metabolites between the different groups. R^2^Y (cum) and Q^2^ (cum) are commonly utilized to describe the level of model fit. In the negative ion mode, the K and M groups R^2^Y and Q^2^ were 0.960 and 0.859, and the M and WH groups were 0.990 and 0.934; In the positive ion mode, the K and M groups R^2^Y and Q^2^ were 0.971 and 0.865, the M and WH groups were 0.990 and 0.889. Moreover, the validity of this model was confirmed by 200 permutation tests. The results presented demonstrate a well-fitting rate of the current model. In addition, S-plot diagrams were utilized to identify differential metabolites (Figure 9).

#### 3.5.2 Identification of potential metabolites

**FIGURE 10:**
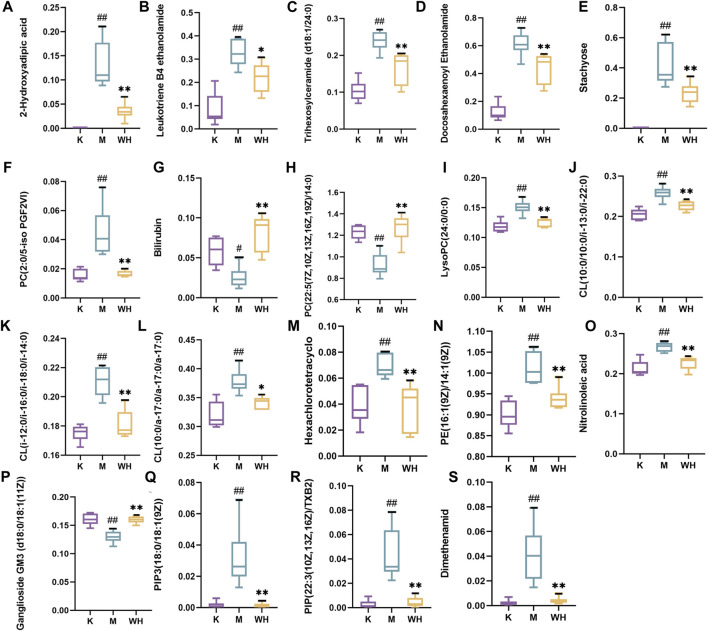
Change trend of different metabolites among the K, M, and WH groups. **(A)**: 2-Hydroxyadipic acid. **(B)**: Leukotriene B4 ethanolamide. **(C)**: Trihexosylceramide (d18:1/24:0). **(D)**: Docosahexaenoyl Ethanolamide. **(E)**: Stachyose. **(F)**: PC(2:0/5-iso PGF2VI). **(G)**: Bilirubin. **(H)**: PC(22:5(7Z,10Z,13Z,16Z,19Z)/14:0). **(I)**: LysoPC(24:0/0:0). **(J)**: CL(10:0/10:0/i-13:0/i-22:0). **(K)**: CL(i-12:0/i-16:0/i-18:0/i-14:0). **(L)**: CL(10:0/a-17:0/a-17:0/a-17:0). **(M)**: Hexachlorotetracyclo. **(N)**: PE(16:1(9Z)/14:1(9Z)). **(O)**: Nitrolinoleic acid. **(P)**: Ganglioside GM3 (d18:0/18:1(11Z)). **(Q)**: PIP3(18:0/18:1(9Z)). **(R)**: PIP(22:3(10Z,13Z,16Z)/TXB2). **(S)**: Dimethenamid. Data are presented as the mean ± SD (*n* = 6). ^#^ *P* < 0.05, ^##^ *p* < 0.01 compared with the K group; ^*^ *p* < 0.05, ^**^ *p* < 0.01 compared with the M group.

**FIGURE 11:**
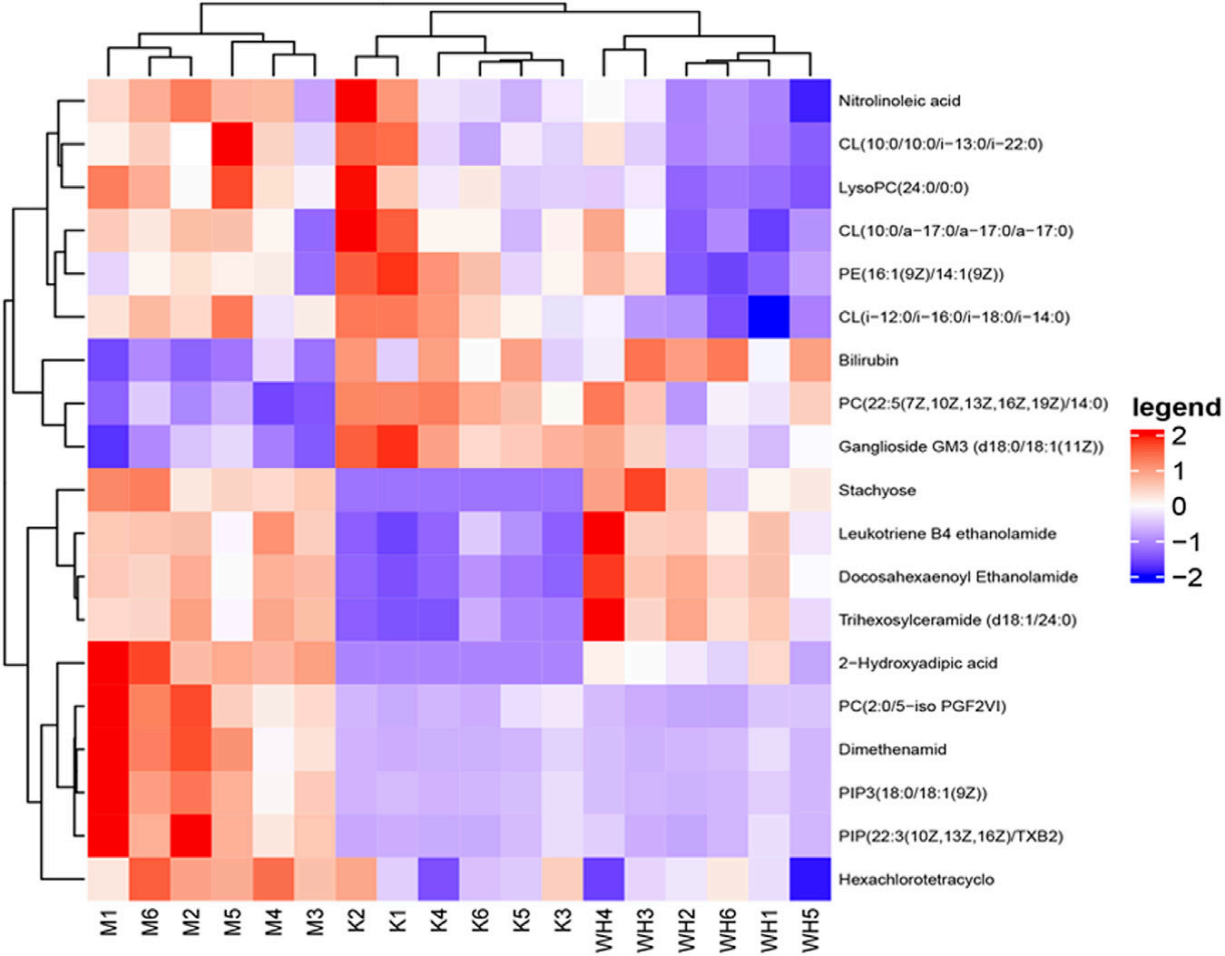
Heatmap of different metabolites among the K, M, and WH groups.

The metabolic meet VIP > 1, |P (corr)| ≥ 0.58, and t-test *p* < 0.05 were identified as potential biomarkers according to OPLS-DA analysis. A total of 19 metabolites were eventually acquired. Subsequently, all metabolites were identified using the HMDB database. Among these, 3 metabolites were down-regulated, and 16 metabolites were up-regulated between the M and K groups. However, the change patterns were reversed between the WH and M groups. The changes in metabolics are displayed in Table 2 and Figure 10. To illustrate the changes in metabolites among the different groups, we created a relevant heatmap (Figure 11).

**TABLE 2:** Metabolites change among different groups.

No	Mode	Metabolism	Error (ppm	RT	*m/z*	Chemical Formula	M *vs* K			WH *vs* M		
							VIP	t-test *p*	Trend	VIP	t-test *p*	Trend
1	ESI-	2-Hydroxyadipic acid	15	0.98	221.07	C_6_H_10_O_5_	1.54	0.000	↑^##^	1.12	0.000	↓^**^
2	-	Leukotriene B4 ethanolamide	9	12.41	1136.82	C_22_H_37_NO_4_	2.11	0.000	↑^##^	1.24	0.013	↓^*^
3	-	Trihexosylceramide (d18:1/24:0)	14	12.71	1180.81	C_60_H_113_NO_18_	1.60	0.000	↑^##^	1.05	0.005	↓^**^
4	-	Docosahexaenoyl Ethanolamide	18	12.76	1112.82	C_24_H_37_NO_2_	3.06	0.000	↑^##^	1.60	0.011	↓^*^
5	-	Stachyose	15	1.33	221.07	C_24_H_42_O_21_	2.75	0.000	↑^##^	1.34	0.017	↓^*^
6	ESI+	PC(2:0/5-iso PGF2VI)	1	8.89	572.30	C_28_H_50_NO_11_P	1.08	0.002	↑^##^	1.17	0.002	↓^**^
7	-	Bilirubin	1	8.40	585.27	C_33_H_36_N_4_O_6_	1.05	0.005	↓^##^	1.68	0.000	↑^**^
8	-	PC(22:5(7Z,10Z,13Z,16Z,19Z)/14:0)	5	8.94	780.55	C_44_H_78_NO_8_P	3.57	0.000	↓^##^	4.32	0.000	↑^**^
9	-	LysoPC(24:0/0:0)	11	9.50	676.46	C_32_H_66_NO_7_P	1.13	0.000	↑^##^	1.11	0.001	↓^**^
10	-	CL(10:0/10:0/i-13:0/i-22:0)	4	10.56	675.96	C_64_H_124_O_17_P_2_	1.46	0.000	↑^##^	1.24	0.003	↓^**^
11	-	CL(i-12:0/i-16:0/i-18:0/i-14:0)	13	10.56	690.48	C_69_H_134_O_17_P_2_	1.24	0.000	↑^##^	1.24	0.000	↓^**^
12	-	CL(10:0/a-17:0/a-17:0/a-17:0)	1	14.36	667.46	C_70_H_136_O_17_P_2_	1.51	0.000	↑^##^	1.28	0.003	↓^**^
13	-	(1S,2R,3S,6S,7S,8S)-1,8,9,10,11,11-Hexachlorotetracyclo[6.2.1.13,6.02,7]dodeca-4,9-diene	6	14.58	751.77	C_12_H_8_Cl_6_	1.06	0.001	↑^##^	1.26	0.004	↓^**^
14	-	PE(16:1(9Z)/14:1(9Z))	9	14.36	692.48	C_35_H_66_NO_8_P	2.15	0.000	↑^##^	1.77	0.003	↓^**^
15	-	Nitrolinoleic acid	6	14.35	678.47	C_18_H_31_NO_4_	1.47	0.000	↑^##^	1.44	0.000	↓^**^
16	-	Ganglioside GM3 (d18:0/18:1(11Z))	6	9.15	1180.76	C_59_H_108_N_2_O_21_	1.10	0.000	↓^##^	1.25	0.000	↑^**^
17	-	PIP3(18:0/18:1(9Z))	14	8.96	1183.51	C_45_H_88_O_22_P_4_	1.07	0.004	↑^##^	1.19	0.003	↓^**^
18	-	PIP(22:3(10Z,13Z,16Z)/TXB2)	3	8.87	1135.51	C_52_H_90_O_20_P_2_	1.28	0.000	↑^##^	1.40	0.001	↓^**^
19	-	Dimethenamid	16	8.77	614.18	C_12_H_18_ClNO_2_S	1.26	0.001	↑^##^	1.35	0.002	↓^**^

Note: ^#^ *p* < 0.05, ^##^ *p* < 0.01 compared with the K group; *p < 0.05, **p < 0.01 compared with the M group.

#### 3.5.3 Metabolic pathway enrichment

Pathways enrichment of all metabolites via Metaboanalyst 5.0. The results indicated that a total of 7 pathways include Glycerophospholipid metabolism, Linoleic acid metabolism, alpha-Linolenic acid metabolism, Glycosylphosphatidylinositol (GPI)-anchor biosynthesis, Galactose metabolism, Porphyrin and chlorophyll metabolism, Arachidonic acid metabolism involved the regulation of alcohol-induced gastric ulcers. More details are shown in Table 3.

**TABLE 3:** Metabolic pathway enrichment results by MetaboAnalyst 5.0.

No.	Pathway Name	Match Status	*p*	Impact
1	Glycerophospholipid metabolism	3/36	0.000121	0.21631
2	Linoleic acid metabolism	1/5	0.01648	0
3	alpha-Linolenic acid metabolism	1/13	0.042394	0
4	Glycosylphosphatidylinositol (GPI)-anchor biosynthesis	1/14	0.045595	0.00399
5	Galactose metabolism	1/27	0.086429	0.05832
6	Porphyrin and chlorophyll metabolism	1/30	0.095651	0.05288
7	Arachidonic acid metabolism	1/36	0.11387	0

### 3.6 Integrating Network analysis and Metabolomics

**FIGURE 12:**
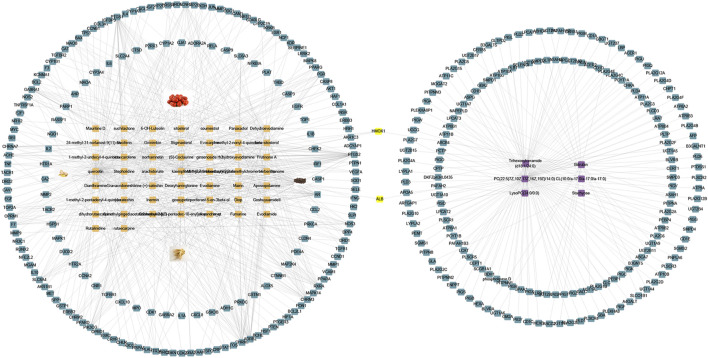
“Compounds-targets-metabolites” network. Blue represent gene, orange represent compounds, purple represent metabolites, and yellow represent common targets.

To further elucidate the possible mechanism between WZYT and potential metabolites, we constructed a “compounds-targets-metabolites” network using Cytoscape 3.9.1 (Figure 12). A total of 173 targets were identified that directly regulate 6 metabolites. Specifically for WZYT, 51 components were found to directly regulate 183 targets. Additionally, two targets, ALB and HMOX1, were identified as common targets involved in both the regulation of metabolism and network analysis.

### 3.7 Western blot analysis

**FIGURE 13:**
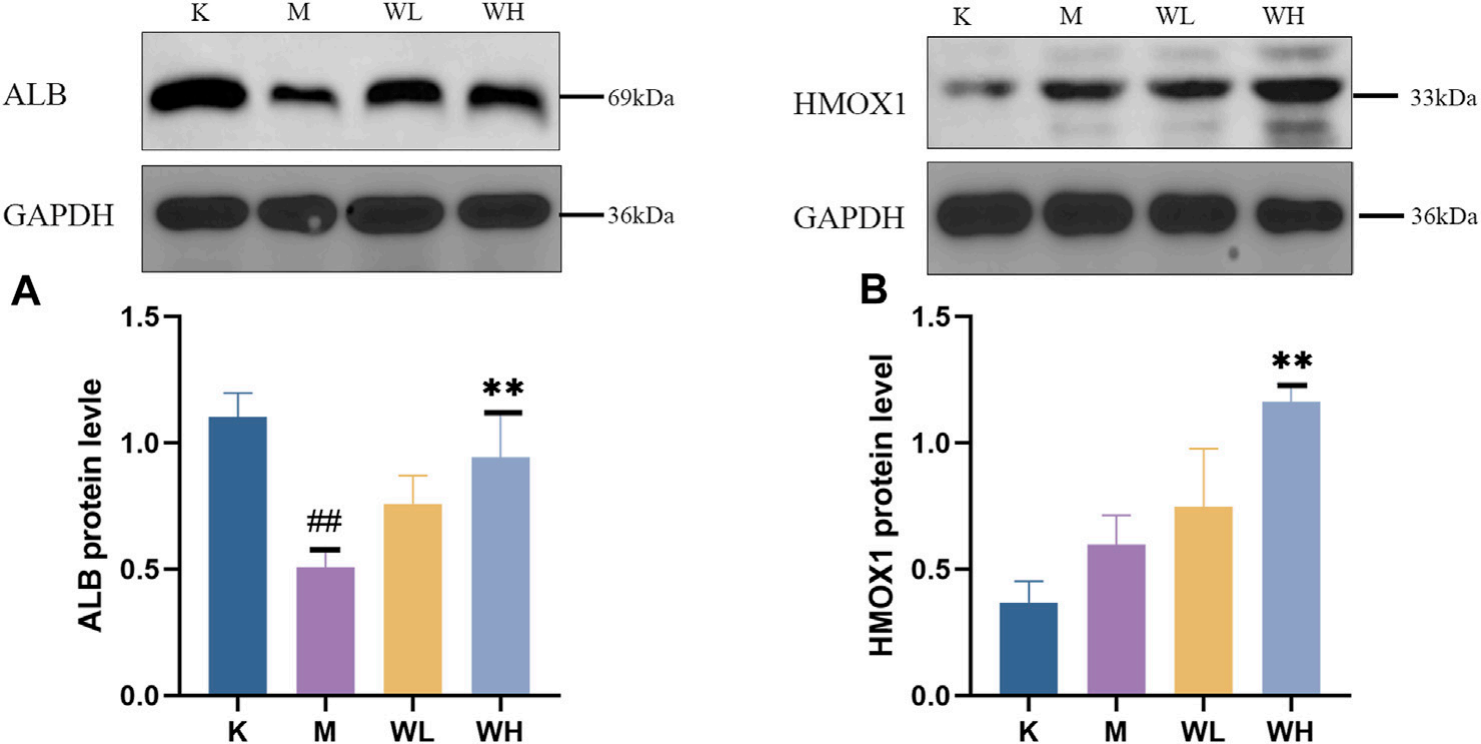
**(A)** The expression of ALB. **(B)** The expression of HMOX1. Data are presented as the mean ± SD (*n* = 3). ^#^ *p* < 0.05, ^##^ *p* < 0.01 compared with the K group; ^*^ *p* < 0.05, ^**^ *p* < 0.01 compared with the M group.

The western blot results revealed that alcohol administration significantly reduced the expression of ALB. However, WZYT administration reversed the ALB decline induced by alcohol (Figure 13A). For the HMOX1, alcohol administration promotes HMOX1 activation as a protective mechanism against oxidative stress damage. WZYT, on the other hand, is capable of enhancing HMOX1 expression to exert its gastrointestinal effects (Figure 13B).

## 4 Discussion

Alcohol-induced stomach ulcers have become one of the most common diseases of the digestive system, and the number is increasing (Zhang et al., 2016a). Compared with Western medicine, Chinese medicine has the characteristics of high safety and good efficacy (Chen et al., 2018). WZYT has been used for thousands of years and has been effective in treating digestive diseases. This study aims to explore the effects and possible mechanisms of Chinese medicine WZYT in alcohol-induced gastric ulcers by network analysis and serum metabolomics.

Alcohol serves as a potent irritant, capable of directly affecting the stomach lining and leading to damage to the gastric mucosa. This impairment often results in bleeding attributed to diminished coagulation processes (Abdel-Kawi et al., 2022). In this study, the gastric mucosa of the M group exhibited features of hemorrhagic erosion and significant loss of integrity. The administration of WZYT effectively alleviated mucosal damage and led to reduced UI levels. HE pathologic staining showed that alcohol administration caused disturbances in the arrangement of the glands and inflammatory cell infiltrate. PAS staining also showed reduced apical staining after alcohol gavage. These phenomena are consistent with previous reports in the literature (Al-Quraishy et al., 2017). The administration of WZYT ameliorated the pathological tissue condition, confirming its efficacy in the protection of gastric mucosa.

Apart from causing damage to the gastric mucosa, alcohol-induced gastric ulcers also prompt the onset of an acute inflammatory response and modify the concentrations of cellular inflammatory factors such as TNF-α, and IL-6 (Salga et al., 2012; Xie et al., 2020). TNF-α is an inflammatory factor secreted by macrophages and plays a pivotal role in activating neutrophils and releasing oxygen-free radicals. The accumulation of TNF-α blocks blood microcirculation and aggravates gastric mucosal damage (Fahmy et al., 2020; Zhou et al., 2021). In addition, the levels of the inflammatory factors of IL-6 also dramatically increased in alcohol-induced gastric ulcers, leading to the breakdown of connexin and further exacerbating gastric mucosal damage (de Souza et al., 2019; Zh et al., 2020). It's worth noting that IL-10 is a typical anti-inflammatory factor in organisms, previous reports have shown that IL-10 inhibits the release of pro-inflammatory factors (Liu et al., 2012). In our study, the levels of TNF-α and IL-6 both increased after alcohol administration, and IL-10 had decreased. WZYT administration could reverse the above indicators, which indicated excellent anti-inflammatory properties.

To delve deeper into the potential mechanisms of WZYT for treating alcoholic gastric ulcers, we conducted an analysis using network analysis. The findings indicate that the PI3K-AKT pathway, which is closely related to lipid metabolism and inflammatory responses, assumes a pivotal role in gastric ulcer treatment (Li et al., 2021b). The pathway of PI3K-AKT has been implicated in apoptosis, cell proliferation and cell migration, which play important roles in cellular epithelial repair, previous literature has also confirmed activation of the PI3K-AKT pathway in gastric ulcers (Tarnawski and Ahluwalia, 2012). The results of the network analysis showed that modulation of the PI3K-AKT pathway may be an important modality for WZYT in the treatment of ethanol-induced gastric ulcers, which should be further validated in the future. Furthermore, serum metabolomics unveiled the engagement of various lipid metabolisms in the modulation of gastric ulcers, such as glycerophospholipid metabolism, linoleic acid metabolism, and arachidonic acid metabolism, which have been reported in previous literature (Zhang et al., 2019; Li et al., 2022b). Lipid metabolism is known to be an important metabolic modality that is regulated by a variety of physiological processes such as cancer, diabetes, atherosclerosis, inflammation, and other autoimmune diseases (Leuti et al., 2020; Yan and Horng, 2020). Alcohol-induced gastric ulcers as a typical inflammatory response actively involved in the regulation of lipid metabolism. LysoPC is a lipid molecule involved in lipid metabolism, stands as a biologically active monoglycerophospholipid with robust pro-inflammatory effects and is closely linked to the enhancement of vascular endothelial permeability, accumulation of this substance leads to severe bleeding in gastric ulcers (Sun et al., 2020; Ren et al., 2021). Our results indicated the level of the lysoPC had an increase in the M group and a decrease in the WH group, confirming the excellent ability of WZYT in the modulation of lipid metabolites.

Integrated network analysis and metabolomics, ALB and HMOX1 were identified as common targets and subsequently validated. ALB is prevalent in humans and many animals, plays a decisive role in maintaining the colloidal osmotic pressure of the blood and transport of various ions (Belinskaia et al., 2021). In addition, due to its distinctive biological structure, ALB acts as an oxygen radical scavenger and demonstrates antioxidant properties (Sitar et al., 2013; Liu et al., 2021). In our study, the level of the ALB had a significant decrease after alcohol administration, which is consistent with previous literature (Zhou et al., 2023). HMOX1 is a prototypical antioxidant gene that safeguards tissues and cells against a range of challenges including ischemia/reperfusion injury, oxidative stress, inflammation, and apoptosis (Otterbein and Choi, 2000; Wagener et al., 2003). The mechanism by which it exerts its antioxidant action is the conversion of oxidants to CO and bilirubin (Uc et al., 2012). It has also been demonstrated that the mutual crosstalk between PI3K-AKT and HMOX1 (Huang et al., 2020). In our research, we confirmed the expression of HMOX1 was activated, and WZYT administration could elevate the expression of HMOX1 to alleviate gastric mucosa injury. Our results are consistent with previous literature (Zhang et al., 2016b).

## 5 Conclusion

The study demonstrated the efficacy of WZYT in enhancing serum biochemical indices and mitigating pathological tissue damage. Network analysis suggests that WZYT may exert a significant impact on alcohol-induced gastric ulcers via the PI3K-AKT pathway, which needs to be further validated in the future. Serum metabolomics highlights the crucial role of lipid metabolism in modulating the progression of alcohol-induced gastric ulcers. The integration of network analysis and metabolomics reveals the multiple pathways and multiple targets that characterize WZYT in the treatment of alcohol-induced gastric ulcers. In the future, we will validate more possible mechanisms such as oxidation, apoptosis, and inflammation. Our research provides novel applications of WZYT, as well as a new strategy for traditional Chinese medicine research.

## Data Availability

The raw data supporting the conclusion of this article will be made available by the authors, without undue reservation.

## Abbreviations

ALB, albumin; BP, biological process; CC, cellular components; DL, drug similarity; GO, Gene ontology; GU, gastric ulcer; H&E, hematoxylin-eosin; HMDB, Human Metabolomr database; HMOX1, heme oxygenase 1; IL-10, interleukin-10; IL-6, interleukin-6; KEGG, Kyoto Encyclopedia of Genes and Genomes; MF, molecular functions; OB, oral bioavailability; OPLS-DA, orthogonal-partial least squares discriminant analysis; PAS, periodic acid-schiff; PCA, principal component analysis; PPI, Protein-protein interaction; TCMSP, Traditional Chinese Medicine Systems Pharmacology database; TNF-α, tumor necrosis factor-α; UI, gastric ulcer index.
